# German Laypeople’s Willingness to Donate Toward Insect Conservation: Application of an Extended Protection Motivation Theory

**DOI:** 10.3389/fpsyg.2021.773913

**Published:** 2022-01-12

**Authors:** Lara Dörge, Milan Büscher, Jasmin Drews, Annike Eylering, Florian Fiebelkorn

**Affiliations:** Department of Biology Didactics, Osnabrück University, Osnabrück, Germany

**Keywords:** insect conservation, donation behavior, protection motivation theory, knowledge, attitudes, intention-behavior gap

## Abstract

It is essential to engage the public in conservation measures to conserve insects. We investigate the Protection Motivation Theory (PMT), as well as knowledge, attitudes, and sociodemographic variables (gender, age, education level, and income) as predictors of willingness to donate (WTD) and actual donations to insect conservation for a representative German sample (*N* = 515; *M_Age_* = 49.36, *SD* = 16.73; female = 50.1%). The PMT subcomponents severity, self-efficacy, and response efficacy, as well as attitudes toward insects, income, and education level, significantly predicted WTD. In contrast, severity, response barriers, age, gender, and the WTD significantly influenced actual donations. Overall, components of the PMT have high predictive power for both dependent variables. Our results suggest that an intention-behavior gap exists between the intention to donate and the actual donation toward insect conservation. Measures to increase WTD and actual donations for insect conservation are discussed.

## Introduction

Insects are the animal class containing the most species and are essential for our ecosystems (Stork, 2018; BfN, 2019). However, 40% of all insect species worldwide are threatened with extinction (Sánchez-Bayo and Wyckhuys, 2019). Researchers expect a progressive loss of insect biomass of 2.5% per year (Sánchez-Bayo and Wyckhuys, 2019). In selected nature reserves in Germany, Hallmann et al. (2017) documented a biomass loss of up to 76% of flying insects over 27 years. Generally, a declining trend in insect populations prevails over both the short and long term (Ries and Nigmann, 2019). These drastic developments are primarily due to anthropogenic influences. For example, the loss of habitats through deforestation, pesticides in intensive agriculture, and urbanization, as well as environmental pollution, and advancing climate change, threaten the survival of insects (Samways, 2018; Cardoso et al., 2020).

The global decline of insects poses a significant challenge because insects perform numerous system-relevant functions in ecosystems (ecosystem services) and are therefore indispensable for humanity (Wilson, 1987). Insects do not only feed other animal species but also ensure the survival of numerous plants as pollinators. Insects also play an essential role in the biological control of organisms, the regulation of energy and nutrient cycles, genetic research, and the provision of medical drugs (Samways, 2018; Segerer and Rosenkranz, 2018; BMU, 2019; Cardoso et al., 2020). In the US insects generate an estimated monetary value of 57 billion US dollars per year through their ecosystem services (Losey and Vaughan, 2006). Nevertheless, the decline of insects is often neglected in politics and media coverage (Snaddon and Turner, 2007; Cardoso et al., 2011, 2020). Several studies show that the willingness of the population to actively engage in insect conservation is low in comparison to larger vertebrates (Martín-López et al., 2007; Prokop and Fančovičová, 2013). This could be because people often perceive insects as unaesthetic, disgusting, or dangerous (Kellert, 1993; Lorenz et al., 2014; Samways, 2018).

The threat to insects is usually not perceived by the general public, and the importance of their ecosystem services is often taken for granted (Martín-López et al., 2007; Cardoso et al., 2011, 2020; Samways et al., 2020). However, since public support for the conservation of insects is crucial, research should analyze psychological factors that influence when, how, and why people engage in insect conservation (Cardoso et al., 2011; Samways, 2018). Since the financial resources available for the preservation of biological diversity are scarce (Lundberg et al., 2019), insect conservation measures often require financial support from the public (Cardoso et al., 2011; Simaika and Samways, 2018). Therefore, identifying environmental psychological factors that influence the willingness to donate (WTD; in literature also expressed as the willingness to pay) and actual donations to insect conservation is of particular importance in this context. This could provide important insights to increase the effectiveness of fundraising campaigns. Several studies have already examined WTD to various environmental and biodiversity conservation measures (e.g., Martín-López et al., 2007; Veríssimo et al., 2009; Wang and Jia, 2012; Kamri, 2013; Batel et al., 2014; Adamu et al., 2015; O’Bryhim and Parsons, 2015; Lundberg et al., 2019). In contrast, only a few studies have examined the actual donation behavior (Srnka et al., 2003; Leliveld and Risselada, 2017). To our knowledge, no studies yet have investigated WTD and actual donations to insect conservation at once.

**Figure 1:**
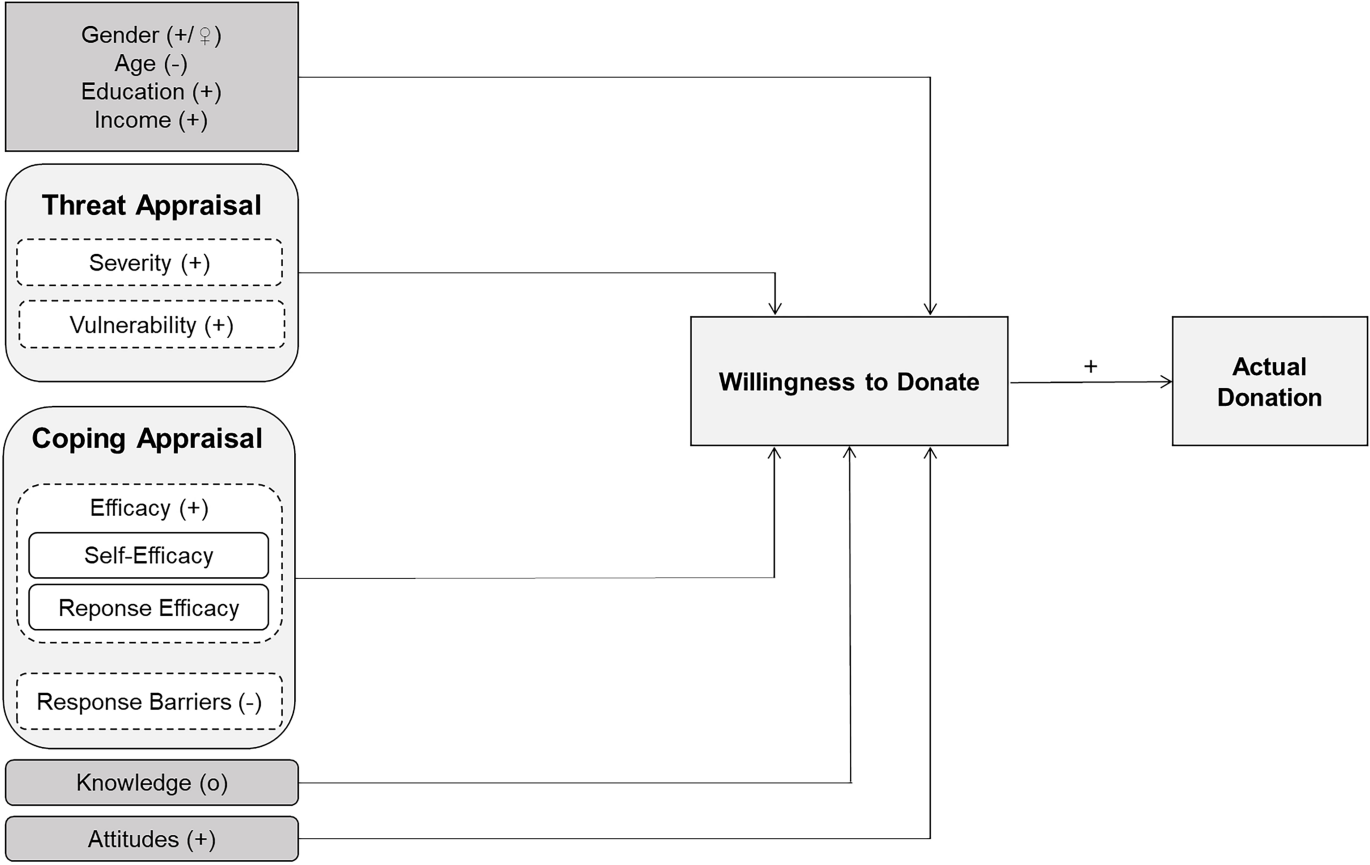
Extended model of the Protection Motivation Theory (PMT) to explain the willingness to donate and actual donations to insect conservation in Germany. Variables of the PMT are colored in light grey. Self-efficacy and response efficacy were summarized as efficacy in this study. Extensions of the PMT are marked in dark gray (gender, age, education, income, knowledge, and attitudes). Latent variables are represented in round boxes and manifest variables are in square boxes. For the sake of clarity, the influences on the actual donations are not shown. It is assumed that the predictors have the same influence on them as on the willingness to donate. Only for attitudes no influence on actual donations is expected. + = positive influence, − = negative influence, o = no influence.

In this study, we use the Protection Motivation Theory (PMT; Rogers, 1983; Rogers and Prentice-Dunn, 1997), as a theoretical model to investigate donation behavior in the context of insect conservation in Germany. This theory has already explained environmentally friendly behavior in several contexts (Kothe et al., 2019). Moreover, multiple studies suggest that additional factors influence WTD and actual donations to biodiversity conservation measures. Sociodemographic characteristics also appear to be important in the present context (e.g., Srnka et al., 2003; Wang and Jia, 2012; Kamri, 2013; Adamu et al., 2015; Leliveld and Risselada, 2017). Additionally, people’s knowledge is often suggested to play a role (Turpie, 2003; Batel et al., 2014; O’Bryhim and Parsons, 2015). Furthermore, attitudes seem to particularly influence the formation of behavioral intentions (Armitage and Christian, 2003; Clayton and Myers, 2009; Ajzen, 2011; Pronello and Gaborieau, 2018) and thereby also the motivation to conserve insects (Cornelisse and Sagasta, 2018). Based on these publications, we expanded our model to include the factors, (1) sociodemographic characteristics, (2) knowledge, and (3) attitudes (Figure 1).

Accordingly, the present study aims to investigate, the components of the PMT, as well as knowledge, attitudes, and sociodemographic variables as possible factors influencing the WTD and actual donations to insect conservation. The findings might be used to develop fundraising campaigns and educational programs to raise public awareness for the conservation of the diversity of insect species [United Nations (UN), 1992].

## Theoretical Background and Current State of Research

### Sociodemographic Variables and Donation Behavior

Although sociodemographic variables usually only explain a small proportion of environmentally friendly behavior (Stern, 2000), studies point toward a non-negligible role of sociodemographic factors in predicting donation behavior. Veríssimo et al. (2009) found that women were more willing to donate to the conservation of endangered bird species. Moreover, women donated slightly more often than men in a study on actual donations to various charity organizations (Leliveld and Risselada, 2017). Regarding age, younger people appear to be more willing to donate to the conservation of biodiversity (Martín-López et al., 2007) and were willing to donate more to charity organizations (Leliveld and Risselada, 2017). In terms of education level, several studies indicate that a higher level of education leads to a higher WTD to the conservation of biodiversity (Wang and Jia, 2012; Kamri, 2013; Adamu et al., 2015) and more donations (Srnka et al., 2003). Similarly, some studies have identified income as a positive predictor of WTD (Ojea and Loureiro, 2007; Wang and Jia, 2012; Kamri, 2013; Adamu et al., 2015). Srnka et al. (2003) also found that participants with a higher income donated more to animal and environmental conservation. In line with these results, we expect that females will be more likely to donate than men, younger people more than older people, higher educated people more than lower educated people, and people with a higher income more than people with a lower income.

### Protection Motivation Theory and Donation Behavior

In several studies, the theoretical assumptions of the PMT successfully helped to predict behaviors and intentions such as the willingness to save energy or engage in climate-friendly behaviors (Kim et al., 2012; Horng et al., 2013; Mankad et al., 2013; Bockarjova and Steg, 2014; Zhao et al., 2016; Rainear and Christensen, 2017; Kothe et al., 2019) as well as the motivation to protect wolves (Hermann and Menzel, 2013).

In the context of this study, we assume that the development of a protection motivation for insects in Germany – i.e., the WTD and actual donations – is based on the two cognitive assessment processes (1) threat appraisal and (2) coping appraisal.

Threat appraisal evaluates both severity and vulnerability. Severity assesses both the perceived severity of the threat to humans and nature as well as ecosystems. Vulnerability is the perceived probability of a threat to affect endangered insect species. Accordingly, the higher people perceive the threat to insects, the more willing they are to implement appropriate conservation behavior.

Coping appraisal, on the other hand, includes self-efficacy and response efficacy as well as perceived response barriers. Self-efficacy is the participants’ belief in their ability to engage in actions counteracting the threat to insect species. Response efficacy assesses the perceived effectiveness of such actions. Thus, a strong coping appraisal is also associated with a stronger protection motivation. However, response barriers negatively influence people’s coping appraisal (Rogers and Prentice-Dunn, 1997; Hermann and Menzel, 2013; Figure 1). These include anticipated barriers to the conservation of endangered insect species, such as adverse consequences for individual stakeholders. In this study, we assume that severity, vulnerability, self-efficacy, and response efficacy positively influence the WTD and actual donations to insect conservation, whereas we expect a negative impact of response barriers.

### Knowledge and Donation Behavior

Knowledge about biodiversity or climate change is often suggested as an important predictor of environmentally friendly behavior by conservationists (Frick et al., 2004). Some studies indeed suggest a positive impact of knowledge on the WTD to the conservation of endangered animal species (Turpie, 2003; Batel et al., 2014; O’Bryhim and Parsons, 2015). However, Onel and Mukherjee (2016) could not find any direct influence of knowledge of ecological facts on the WTD to environmental conservation and factual knowledge generally does not appear to influence environmental behavior (Frick et al., 2004). Accordingly, we assume that factual knowledge about insects does not influence WTD or actual donations (Figure 1).

### Attitudes and Donation Behavior

Environmental attitudes directly and indirectly influence environmental conservation behavior *via* behavioral intentions (Fishbein and Ajzen, 1975; Ajzen, 1991; Pronello and Gaborieau, 2018). Several studies have already demonstrated a positive impact of environmental attitudes on the WTD to the conservation of various endangered species (Kotchen and Reiling, 2000; Aldrich et al., 2006; Spash et al., 2009; Wilson and Bruskotter, 2009; Choi and Fielding, 2013; Zander et al., 2014). However, invertebrates, which include insects, were only considered in the study by Martín-López et al. (2007) who found that people tend to have unfavorable attitudes toward them. Many people perceive insects as unaesthetic (Kellert, 1993; Lorenz et al., 2014; Samways, 2018), which could result in a lowered willingness to conserve these animals (Martín-López et al., 2007). As such, we assume that positive attitudes toward insects positively influence the WTD and actual donations toward insect conservation in Germany.

## Materials and Methods

### Data Collection and Sampling

Data were collected with a questionnaire sent to participants throughout Germany in August 2019 *via* the online access panel of Consumerfieldwork GmbH. At the time, the Panel Book of Consumerfieldwork listed 49,480 respondents in Germany with a minimum age of 18 (Consumerfieldwork GmbH, 2018). Upon completion of the entire questionnaire, the participants received 2 €, which they could donate to an insect conservation project of the NABU (German Nature and Biodiversity Conservation Union) (2019).

The final sample consisted of 515 participants. As the present research was embedded in a larger project, statistical power could only be estimated *post hoc*. However, our statistical power was sufficient to detect at least medium effect sizes (*f^2^* = 0.15). It was aimed to achieve a sample representative of the total German population (Table 1). Therefore, age, gender, and federal-state served as stratification criteria. The gender distribution of the sample was 50.1% women and 49.9% men, almost identical to the German population [50.7% female, 49.3% male; Destatis (Federal Bureau of Statistics), 2019b]. The participants’ age ranged from 18 to 91 years, with a mean value of 49.36 (*SD* = 16.73); higher than the mean of 44.4 years for the entire German population [Destatis (Federal Bureau of Statistics), 2019a]. In addition, the sample had an above-average level of education compared to the German population as a whole [Destatis (Federal Bureau of Statistics), 2020; see Table 1]. The average monthly net household income of the participants was 2,500–2,750 €, which is below the German average net household income of 3,399 € [Destatis (Federal Bureau of Statistics), 2019b].

**Table 1:** Frequency statistics of the sociodemographic variables of the participants (*N* = 515).

Variable	Answer format	Frequency in sample
Gender	“male” (0)	49.9%
	“female” (1)	50.1%
Age	Open question	18–20 years = 1.2%
		21–24 years = 5.1%
		25–39 years = 26.8%
		40–59 years = 34.9%
		60–64 years = 10.4%
		≥ 65 years = 20.8%
Educational level	“No school leaving certificate” (1)	0.0%
	“Secondary school certificate” (2)	11.3%
	“Intermediate secondary school certificate” (3)	32.6%
	“Advanced technical college entrance qualification” (4)	11.3%
	“General qualification for university entrance” (5)	44.9%
Income^1^	“not specified”	
	“less than 150 €” (1)	0.9%
	“150–450 €” (2)	0.9%
	“451–850 €” (3)	3.6%
	“851 to less than 1.000” (4)	4.1%
	“1.000 to less than 1.250 €” (5)	6.2%
	“1.250 to less than 1.500 €” (6)	4.7%
	“1.500 to less than 1.750 €” (7)	6.4%
	“1.750 to less than 2.000 €” (8)	6.6%
	“2.000 to less than 2.250 €” (9)	6.0%
	“2.250 to less than 2.500 €” (10)	6.6%
	“2.500 to less than 2.750 €” (11)	5.8%
	“2.750 to less than 3.000 €” (12)	6.8%
	“3.000 to less than 3.250 €” (13)	6.0%
	“3.250 to less than 3.500 €” (14)	5.8%
	“3.500 to less than 3.750 €” (15)	5.1%
	“3.750 to less than 4.000 €” (16)	6.0%
	“4.000 to less than 4.500 €” (17)	6.6%
	“4.500 to less than 5.000 €” (18)	4.9%
	“5.000 to less than 5.500 €” (19)	3.0%
	“5.500 to less than 6.000 €” (20)	0.9%
	“6.000 to less than 7.500 €” (21)	1.1%
	“7.500 to less than 10.000 €” (22)	1.3%
	“10.000 to less than 20.000 €” (23)	0.6%
	“20.000 € and more” (24)	0.4%

The sociodemographic variables were collected according to the specifications of the Destatis (Federal Bureau of Statistics) (2016).

1 46 participants selected the “no information” option.

### Questionnaire and Variables

The questionnaire consisted of 60 items, arranged in the following order: (1) attitudes toward insects, (2) PMT constructs (threat and coping appraisal), (3) knowledge about insects, (4) WTD to insect conservation, (5) donation to insect conservation, and (6) sociodemographic data. An attention check formulated as “*Please click on the far left on ‘completely disagree’”* was included. Participants who failed this check were excluded.

#### Willingness to Donate and Actual Donations

Both WTD and actual donations to insect conservation in Germany were measured in the present study. WTD to insect conservation was inquired with the item “*I would donate money for projects that actively support the conservation of endangered insect species in Germany*,” following Büssing et al. (2018). Participants could answer on a six-point Likert scale from 1 = “*completely disagree*” to 6 = “*completely agree*.” The participants’ actual donation behavior was measured using a stepless slider. They could donate a freely selectable percentage of their 2 € reward, to a specified insect conservation project of the NABU. WTD and the actual donations were surveyed on separate pages of the questionnaire. Participants could not adjust their WTD after making their selection and were not informed that they would be asked about their actual donation. This allowed to test for the frequently cited intention-behavior gap (Sheeran and Webb, 2016) between behavioral intentions (WTD) and actual behavior (actual donations) in the context of insect conservation in Germany.

Several studies have already examined the WTD to various environmental and biodiversity conservation measures (e.g., Martín-López et al., 2007; Veríssimo et al., 2009; Wang and Jia, 2012; Kamri, 2013; Batel et al., 2014; Adamu et al., 2015; O’Bryhim and Parsons, 2015; Lundberg et al., 2019). Since measuring actual environmental behavior is associated with many temporal, financial, and measurement constraints (Steg and de Groot, 2019), only a few studies have examined the actual donations to environmental conservation organizations. For example, Leliveld and Risselada (2017) found that 89% of the respondents did not donate their reward of 0.67 € to environmental organizations.

We assume that WTD will have a positive influence on the actual donations to insect conservation in Germany (Figure 1). In line with the Theory of Planned Behavior, a strong behavioral intention is positively related to the actual behavior (Fishbein and Ajzen, 1975; Ajzen, 1991; Pronello and Gaborieau, 2018) and we expect this to translate to our studied context.

#### Sociodemographic Variables

The sociodemographic variables gender, age, educational level (highest level of secondary education), and income (monthly net household income) were included in the analyses of this study (for frequency data, see Table 1; for descriptive statistics, see Table 2). The gender was recorded dichotomously (0 = “*male*,” 1 = “*female*”) and age as a whole number. Education level and income were assigned to the appropriate category according to the specifications of the Federal Bureau of Statistics [Destatis (Federal Bureau of Statistics), 2016]. Participants could select “*no information*,” these cases were subsequently treated as missing values.

**Table 2:** Overview of Spearman bivariate correlations and descriptive statistics of the collected variables (*N* = 515).

Variable	(1)	(2)	(3)	(4)	(5)	(6)	(7)	(8)	(9)	(10)	(11)	(12)
(1) Gender^1^	-											
(2) Age	−0.11^*^	-										
(3) Educational level	0.02	−0.35^***^	-									
(4) Income^2^	−0.15^**^	−0.21^***^	0.30^***^	-								
(5) Severity^3^	−0.05	0.14^**^	−0.12^**^	−0.10^*^	-							
(6) Vulnerability^4^	−0.12^**^	−0.02	0.01	0.05	−0.11^*^	-						
(7) Efficacy^3^	−0.09^*^	0.04	−0.06	−0.02	0.63^***^	−0.19^***^	-					
(8) Response barriers^3^	0.07	−0.10^*^	−0.05	0.11^*^	−0.32^***^	0.02	−0.50^***^	-				
(9) Knowledge	−0.22^***^	−0.05	−0.14^**^	0.15^**^	0.15^**^	0.04	0.17^***^	−0.12^**^	-			
(10) Attitudes^5^	−0.21^***^	0.19^***^	−0.02	−0.04	0.54^***^	−0.06	0.53^***^	−0.34^***^	0.24^***^	-		
(11) Willingness to donate^3^	−0.09^*^	−0.03	0.11^*^	0.13^**^	0.38^***^	−0.10^*^	0.38^***^	−0.18^***^	0.13^**^	0.47^***^	-	
(12) Donation^6^	0.09^*^	−0.14^**^	0.04	0.05	0.24^***^	0.03	0.23^***^	−0.15^**^	0.06	0.17^***^	0.36^***^	-
Items	1	1	1	1	6	3	6	4	12 (16)	23 (24)	1	1
Mean value	-	49.36	-	-	5.18	3.33	4.62	2.74	6.16	3.46	3.71	28.25
Standard Deviation	-	16.73	-	-	0.85	0.82	0.83	0.99	2.20	0.68	1.30	33.70
Median	-	50.00	-	-	5.33	3.33	4.67	2.75	6.00	3.52	4.00	10.00
Skewness	-	−0.03	-	-	−1.41	−0.07	−0.25	0.27	−0.24	−0.39	−0.59	0.99
Kurtosis	-	−1.15	-	-	2.77	0.50	−0.51	−0.21	−0.43	−0.32	−0.01	−0.24
K-S test	-	0.09^***^	-	-	0.18^***^	0.14^***^	0.07^***^	0.06^***^	0.13^***^	0.06^***^	0.25^***^	0.23^***^

A significant Kolmogorov-Smirnov test (K-S test) indicates non-normally distributed data (Field, 2018). In the items row, the number of items of the original scale is given in parentheses. This number indicates the number of items for the knowledge and attitude variables before items were selected based on item difficulty (knowledge) or principal component analysis (attitudes).

1 0 = male; 1 = female.

2 46 participants selected the “no information” option. This was treated as a missing value (*N* = 469).

3 Six-point Likert scale: 1 = “completely disagree” to 6 = “completely agree.”

4 Six-point Likert scale: 1 = “very unlikely” to 6 = “very likely.”

5 Five-point Likert scale: 1 = “completely disagree” to 5 = “completely agree.”

6 Sliding scale: 0–100%.

* *p* < 0.05,

** *p* < 0.01,

*** *p* < 0.001.

#### Threat Appraisal and Coping Appraisal

The survey of threat and coping appraisal was based on that of Hermann and Menzel (2013). Items were adapted to the context of the present study by replacing the term “*animal*” with “*endangered insect species*.” Threat appraisal was measured with severity (six items) and vulnerability (three items). Example items are: “*If endangered insect species are not preserved in Germany, it would be bad for future generations*” (severity), “*The populations of various endangered insect species in Germany will recover*” (vulnerability; reverse coded item). Self-efficacy (three items), response efficacy (three items), and response barriers (four items) were measured for the coping appraisal (see Table 2 for answer format). Example items are: “*I can help prevent the extinction of endangered insect species in Germany*” (self-efficacy), “*There are initiatives that can ensure the survival of endangered insect species in Germany*” (response efficacy), “*Farmers’ crop losses argue against protecting endangered insect species*” (response barriers). Inversely formulated items were recoded.

The factor loadings of the items of the PMT constructs were analyzed using principal component analysis. For the threat appraisal items, two factors could be extracted in line with the PMT. Severity explained 50.6% and vulnerability explained 27.9% of the total explained variance of 78.5%. The reliability analyses with Cronbach’s *α* values of 0.90 (vulnerability) and 0.93 (severity) showed high internal consistency of both constructs (Field, 2018).

For the coping appraisal items, only two factors instead of three were extracted. The items of self-efficacy and response efficacy, with factor loadings >0.4, loaded on a single dimension (Field, 2018). Based on these results, the components self-efficacy and response efficacy were combined to one efficacy dimension of coping appraisal for the remaining analyses. In a comparable study, Mankad et al. (2013) also analyzed the two constructs as a combined unidimensional measure of efficacy based on a high correlation between the efficacy constructs (*r* = 0.66; *p* < 0.001). Overall, efficacy explained 33.1% and response barriers explained 24.3% of the total explained variance of 57.4%. Cronbach’s *α* values of 0.69 (response barriers) and 0.85 (efficacy) indicate sufficient reliability of the constructs (Field, 2018).

#### Knowledge

The knowledge test aimed to inquire broad knowledge about insects based on the knowledge categories by Kellert (1993). We used a total of 16 items for the following seven knowledge categories: (1) Biological characteristics of insects (seven items), (2) populations and vulnerability of insects (three items), (3) insects in agriculture and horticulture (two items), (4) taxonomy of insect species (one item), (5) insects related to human diseases (one item), (6) bees (one item), and (7) spiders as “non-insects” (one item).

Based on knowledge questions of Kellert (1993) about invertebrates and knowledge questions of Prokop and Tunnicliffe (2008) about spiders we developed true or false statements for these categories. An example item of the test is: “*Insects make up around 70% of the world’s animal species*.” The participants’ answers were coded as 0 = “*wrong*” and 1 = “*right*.” “*Do not know*” answers were coded as wrong.

Of the 16 total items, we selected 12 based on their item difficulty (Moosbrugger and Kelava, 2012). Accordingly, too simple knowledge questions that were answered correctly by more than 80% of the participants were excluded from further analysis (a total of four items). No question was answered correctly by less than 20% of the participants; thus, no too difficult knowledge questions could be identified. With each correct answer, the participants received one point so that a maximum value of 12 points could be reached. A high test score was interpreted as higher insect knowledge.

#### Attitudes

The survey of attitudes toward insects was based on Prokop et al. (2009) as well as Prokop and Tunnicliffe (2008) who developed a scale for attitudes toward spiders. The original items were adapted to the present study by replacing the term “*spider*” with “*insect*.” Furthermore, some items on the characteristic features of spiders were reworded to account for the specific properties of insects. For example, “*I would like to know more about the weaving behavior of orb weaver spiders.*” (Prokop et al., 2009) became the item *“I would like to know more about the flight behavior of insects*.”

A total of 24 items was used to survey the general attitudes toward insects (see Table 2 for answer format). Inversely formulated items were recoded. These attitudes can theoretically be divided into the four dimensions (1) scientistic, (2) ecologistic, (3) negativistic, and (4) naturalistic (Kellert, 1993; Prokop and Tunnicliffe, 2008; Prokop et al., 2009). Example items were: *“I would like to know more about tropical insect species*.” (scientistic), “*Insects should receive more attention*.” (ecologistic), “*I get nervous when someone tells me that there is an insect near me*.” (negativistic), and “*I would like to catch an insect with my bare hands.*” (naturalistic). A principal component analysis could extract the four theoretically postulated dimensions. However, the assumed distribution of some items was slightly adjusted due to cross-loading by items that overlapped in content. Based on Prokop et al. (2009), we ultimately combined all items of the four dimensions to form a general scale for attitudes toward insects. For our modified scale, we obtained a Cronbach’s *α* value of 0.93 indicating high internal consistency (Field, 2018).

### Statistical Analysis

All statistical analyses were performed using IBM^©^ SPSS^©^ Statistics (version 26.0) software. In the first step, principal component analyses with Varimax rotation were performed to check the variables for dimensionality. Beforehand, the suitability of the data for factor analysis was tested using the Kaiser-Meyer-Olkin criterion. All values were above 0.80; thus, the sampled data appeared to be well suited (Field, 2018). In addition, the reliability of the scales was analyzed using Cronbach’s *α* analyses. The data were then tested for normal distribution using a graphical analysis of the Q-Q plots, the Kolmogorov-Smirnov test, and the skewness and kurtosis (Field, 2018; Table 2). As none of the collected variables were normally distributed, we subsequently used robust, non-parametric tests for the statistical analyses when appropriate. To identify correlations between the variables, a Spearman correlation analysis was performed (Table 2). The influence of the predictors on WTD and the actual donations toward insect conservation in Germany was analyzed with a multiple hierarchical regression for both variables (Tables 3, 4).

**Table 3:** Results of multiple hierarchical regression on the influence of the predictors on willingness to donate and actual donations to insect conservation (*N* = 469).

Willingness to donate				Actual donations			
	Variable	*b*	*SE b*	*β*	Variable	*b*	SE b	*β*
Model 1	Constant	3.11^***^	0.37		Constant	32.04^**^	9.63	
	Gender	−0.24	0.12	−0.09	Gender	5.83	3.17	0.09
	Age	0.00	0.00	0.02	Age	−0.17	0.10	−0.08
	Education	0.10	0.06	0.08	Education	−0.16	1.54	−0.01
	Income	0.03	0.01	0.10	Income	0.23	0.33	0.04
Model 2	Constant	−0.96	0.67		Constant	−21.56	19.09	
	Gender	−0.16	0.11	−0.06	Gender	7.64^*^	3.05	0.11
	Age	−0.00	0.00	−0.02	Age	−0.24^*^	0.10	−0.12
	Education	0.12^*^	0.05	0.10	Education	−0.36	1.49	−0.01
	Income	0.03^*^	0.01	0.11	Income	0.40	0.32	0.06
	Severity	0.47^***^	0.08	0.31	Severity	8.35^***^	2.27	0.21
	Vulnerability	−0.07	0.07	−0.04	Vulnerability	2.24	1.87	0.06
	Efficacy	0.37^***^	0.09	0.23	Efficacy	3.01	2.57	0.07
	Response barriers	0.03	0.06	0.03	Response barriers	−3.60^*^	1.75	−0.11
Model 3	Constant	−1.47^*^	0.65		Constant	−21.49	19.32	
	Gender	−0.00	0.11	−0.00	Gender	7.58^*^	3.18	0.11
	Age	−0.01	0.00	−0.07	Age	−0.24^*^	0.10	−0.12
	Education	0.10^*^	0.05	0.09	Education	−0.30	1.50	−0.01
	Income	0.03^**^	0.01	0.11	Income	0.42	0.32	0.06
	Severity	0.30^***^	0.08	0.19	Severity	8.18^**^	2.40	0.21
	Vulnerability	−0.07	0.06	−0.05	Vulnerability	2.28	1.87	0.06
	Efficacy	0.21^*^	0.09	0.14	Efficacy	2.86	2.66	0.07
	Response barriers	0.07	0.06	0.05	Response barriers	−3.58^*^	1.76	−0.11
	Knowledge	−0.01	0.02	−0.02	Knowledge	−0.40	0.72	−0.03
	Attitudes	0.68^***^	0.10	0.36	Attitudes	1.09	2.87	0.02

Willingness to donate

Model 1: adj. *R*^2^ = 0.022; Δ*R*^2^ = 0.022; *p* < 0.01.

Model 2: adj. *R*^2^ = 0.256; Δ*R*^2^ = 0.234; *p* < 0.001.

Model 3: adj. *R*^2^ = 0.326; Δ*R*^2^ = 0.070; *p* < 0.001.

* *p* < 0.05,

** *p* < 0.01,

*** *p* < 0.001.

Actual donations

Model 1: adj. *R*^2^ = 0.008; Δ*R*^2^ = 0.008; *p* > 0.05.

Model 2: adj. *R*^2^ = 0.096; Δ*R*^2^ = 0.088; *p* < 0.001.

Model 3: adj. *R*^2^ = 0.093; Δ*R*^2^ = −0.003; *p* < 0.001.

**Table 4:** Additional step of the multiple hierarchical regression including the influence of willingness to donate on actual donations to insect conservation (*N* = 469).

Variable	Actual donations		
	*b*	*SE b*	*β*
Constant	−9.85	18.75	
Gender	7.59^*^	3.07	0.11
Age	−0.20^*^	0.10	−0.10
Education	−1.10	1.46	−0.04
Income	0.19	0.31	0.03
Severity	5.84^*^	2.35	0.15
Vulnerability	2.86	1.81	0.07
Efficacy	1.18	2.59	0.03
Response barriers	−4.13^*^	1.70	−0.12
Knowledge	−0.29	0.70	−0.02
Attitudes	−3.26	2.91	−0.09
WTD	7.92^***^	1.35	0.31

Actual donations

adj. *R*^2^ = 0.155; Δ*R*^2^ = 0.062; *p* < 0.001.

* *p* < 0.05,

*** *p* < 0.001.

## Results

### Descriptive Statistics

Descriptive statistics about the variables inquired can be obtained from Table 2. Of the 515 total participants 319 (61.9%) donated at least 1% of their reward (169 of all females, 65.5%, 140 of all males, 58.4%). Overall females donated 7.6% more than males to endangered insects in Germany which is statistically significant (*t*(513) = −2.16, *p* < 0.01). Furthermore, 221 (42.9%) of all participants donated at least 25% of their reward, 186 (36.1%) at least 50% and 59 (11.5%) 100%.

While the mean score for knowledge was 6.16 (*SD* = 2.20) none of the participants achieved the highest possible score of 12. In total, two participants did not answer any question correctly.

### Spearman Correlation Analysis

Table 2 shows the correlations between all collected variables. Almost all independent variables showed significant correlations with the WTD; except for age. The highest correlations were observed between WTD and attitudes (*r* = 0.47; *p* < 0.001), severity (*r* = 0.38; *p* < 0.001) as well as efficacy (*r* = 0.38; *p* < 0.001). With the exception of education, income, vulnerability, and knowledge, all variables significantly correlated with the actual donation. The highest correlations were between donation and WTD (*r* = 0.36; *p* < 0.001), severity (*r* = 0.24; *p* < 0.001) as well as efficacy (*r* = 0.23; *p* < 0.001).

### Multiple Hierarchical Regression

Overall, five of the 10 analyzed predictors showed a significant influence on WTD in the complete regression model (Table 3). Educational level (*β* = 0.09; 95% CI, 0.002–0.20; *p* < 0.05), income (*β* = 0.11; 95% CI, 0.01–0.05; *p* < 0.01), severity (*β* = 0.19; 95% CI, 0.14–0.45; *p* < 0.001), efficacy (*β* = 0.14; 95% CI, 0.04–0.39; *p* < 0.05), and attitudes (*β* = 0.36; 95% CI, 0.49–0.86; *p* < 0.001) were identified as positive predictors. Attitudes had by far the strongest influence on the WTD.

The inclusion of the sociodemographic variables in the first step of the multiple hierarchical regression explained 2.2% of the total variance [*F*(4, 464) = 3.66, *p* < 0.01]. In a second step, 23.4% of the variance could additionally be explained by adding the PMT constructs [*F*(8, 460) = 21.08, *p* < 0.001]. An additional 7.0% of the variance could be explained by including the variables knowledge and attitudes in the third step of the regression [*F*(10, 458) = 23.62, *p* < 0.001]. Thus, the model can explain a total of 32.6% of the variance in WTD to insect conservation in Germany.

Four of the 10 tested predictors significantly influenced the actual donations (Tables 3, 4). While being a female (*β* = 0.11; 95% CI, 1.34–13.21; *p* < 0.05) and severity (*β* = 0.21; 95% CI, 3.47–12.99; *p* < 0.01) were identified as positive predictors, the variables age (*β* = −0.12; 95% CI, −0.44 to −0.05; *p* < 0.05) and response barriers (*β* = −0.11; 95% CI, −7.03 to −0.13; *p* < 0.05) had a negative influence. Severity had the strongest influence on the actual donation.

By including the sociodemographic variables in the first step of a multiple hierarchical regression, 0.8% of the total variance could be explained [*F*(4, 464) = 1.96, *p* = 0.099]. A further 8.8% of the variance could be explained by including the PMT constructs in the second step of the regression [*F*(8, 460) = 7.22, *p* < 0.001]. No further variance could be resolved by adding the variables knowledge and attitudes in the third step [*F*(10, 458) = 5.80, *p* < 0.001]. Thus, the model could explain 9.3% of the variance in the actual donation toward insect conservation in Germany.

To investigate the additional influence of WTD as an independent variable on the actual donation, an additional fourth regression step was performed (Table 4). In this analysis, the same predictors gender (*β* = 0.11; 95% CI, 1.57–13.62; *p* < 0.05), age (*β* = −0.10; 95% CI, −0.39 to −0.14; *p* < 0.05), severity (*β* = 0.15; 95% CI, 1.22–10.46; *p* < 0.01), and response barriers (*β* = −0.12; 95% CI, − 7.46 to −0.79; *p* < 0.05) showed a significant impact on donation. However, WTD had the greatest influence on actual donations (*β* = 0.31; 95% CI, 5.27–10.57; *p* < 0.001) and could explain a further 6.2% of the variance. Overall, a total of 15.5% of the variance in the actual donations to insect conservation in Germany could be explained by our predictors. In total, 469 participants entered the regression models, the other participants were excluded because they did not give information for at least one of the variables.

## Discussion

### Willingness to Donate and Actual Donations to Insect Conservation in Germany

Willingness to donate was rather high in the present study as the mean (3.71) is above 3, the mid-point of the scale. Actual donations on the other hand were rather low (*M* = 28.25) compared to the mid-point of 50%, however, there was high variability between the participants (*SD* = 33.70). In this study, WTD had a significant positive influence on the actual donation to insect conservation in Germany, which was in line with our hypothesis. Nevertheless, it must be taken into account that the WTD could only explain 6.2% of the total variance of the actual donations. In addition, some predictors (educational level, income, attitude, and efficacy) showed a significant impact on WTD, but not on actual donations. These results serve as evidence for the frequently described intention-behavior gap (Ajzen et al., 2004; Sheeran and Webb, 2016). It appears that there is a discrepancy between the expressed behavioral intention and the actual execution of that behavior. According to this phenomenon, some participants might have indicated a high WTD to insect conservation due to social desirability because they did not expect the subsequent question to be an opportunity to actually donate. Literature suggests that this could be avoided by asking participants about concrete ways to implement their behavior (Gollwitzer, 1999). In line with this, future research should specifically investigate factors that inhibit and promote the connection between conservation intention and action to derive context-specific strategies for promoting actual donations to insect conservation.

Although Martín-López et al. (2007) report that people were less willing to donate to insects compared to mammals and plants, participants in our study had a positive WTD toward insect conservation relative to the center of our measurement scale. The participants in this study donated more frequently and larger amounts compared to those in the study by Leliveld and Risselada (2017). Thus, it appears that there is some willingness in members of the German public to donate to insect conservation.

### Influence of the Studied Predictors

#### Sociodemographic Variables

Overall, the sociodemographic variables could only explain 2.2% of the total variance in WTD and 0.8% of the total variance in actual donations to insect conservation in Germany. These results support the assumption that sociodemographic factors explain only a small proportion of environmentally friendly behavior (Stern, 2000). Although gender did not influence the WTD, in line with our hypothesis women donated significantly more of the 2 € to NABU’s insect conservation project than men did (7.6%). Similar results have been found for donations toward various non-profit organizations (Leliveld and Risselada, 2017). Women may have more positive attitudes toward insect conservation than men, which could explain this discrepancy (Fančovičová and Prokop, 2017; Penn et al., 2018). However, correlational evidence from our study suggests that men have more positive attitudes toward insects in general (*r* = −0.21; *p* < 0.001). Nevertheless, it may make sense to develop gender-specific fundraising campaigns for biodiversity conservation, especially in the context of insects.

In addition, age influenced only the actual donation behavior, with younger participants donating more to insect conservation than older participants. This echoes the results of the study by Leliveld and Risselada (2017), who found that older people donated less to charity than younger people. Notably, in this study, there was a negative correlation between age and income (*r* = −0.21; *p* < 0.001). Thus, the lower income of the older participants in this sample could explain their lower donations.

Both the level of education and income could significantly predict the WTD to insect conservation. A high level of education, as well as a higher income, positively influenced the WTD. These results are consistent with the findings of several studies on the WTP for the conservation of biodiversity (Wang and Jia, 2012; Kamri, 2013; Adamu et al., 2015). However, in contrast to the results of the study by Srnka et al. (2003) on donations to different non-profit organizations, our results suggest neither education nor income influenced actual donations. Future studies should therefore shed more light on the previously described discrepancy between WTD and donation for insect conservation.

#### Protection Motivation Theory Constructs

While severity and efficacy were identified in this study as positive predictors of WTD to insect conservation, the constructs vulnerability and response barriers were not significant. For actual donations, this changed as response barriers had a negative influence while severity had a positive impact. Accordingly, our hypotheses that all constructs of the PMT would explain WTD and actual donations could only be partially confirmed. Nevertheless, the PMT constructs explained most of the overall variance in WTD and actual donation compared to the other tested variables. Thus, the PMT made a significant contribution to the understanding of psychological factors influencing the WTD and actual donations to insect conservation.

Of the PMT constructs, severity had the strongest positive influence on the WTD and actual donations to insect conservation. Several studies also found that high levels of severity positively influenced the willingness to engage in environmentally-friendly behaviors (Kim et al., 2012; Mankad et al., 2013; Bockarjova and Steg, 2014; Rainear and Christensen, 2017) and the motivation to support the return of wolves to Germany (Hermann and Menzel, 2013). Chen (2015) and Huth et al. (2018) were able to increase perceived severity and in turn also environmentally friendly behavior by creating fear appeals. Thus, in line with previous findings, concerns about the negative impact on the environment and human well-being associated with insect mortality seem to play a significant role in the WTD and actual donations to insect conservation. These findings emphasize that public awareness of the threat and the ecological importance of insects is much needed (Simaika and Samways, 2018; BMU, 2019; Cardoso et al., 2020). Accordingly, educational programs and awareness campaigns should be developed to familiarize people with the essential role of insects and the consequences of their endangerment. In future research, it would be interesting to investigate which measures can increase the perceived severity of the threat to insects to promote the population’s WTD and actual donation to insect conservation in the short and long term.

Contrary to our assumption, vulnerability did not influence WTD or actual donations to insect conservation. Hermann and Menzel (2013), however, raised concerns that inversely worded items potentially raised participants’ confidence about the success of conservation measures (e.g., “*The conservation of many endangered insect species will be successful*.”). Thus, the perceived threat to insects may not have been adequately captured in the present study. Future studies should therefore use a more negative formulation to assess vulnerability (Hermann and Menzel, 2013).

Consistent with other studies (Kim et al., 2012; Hermann and Menzel, 2013; Horng et al., 2013; Mankad et al., 2013; Bockarjova and Steg, 2014; Rainear and Christensen, 2017), the variable efficacy (as a combined measure of self-efficacy and response efficacy) had a positive effect on the WTD to insect conservation in Germany. Previous research that combined self-efficacy and response efficacy into one construct also found a positive influence on pro-environmental intentions (Mankad et al., 2013). These results imply that public understanding of possible protective measures for the conservation of endangered insect species in Germany is important. If people believe in their ability as well as in the effectiveness of specific responses for the conservation of insects, it is more likely that they will support such measures financially. Future fundraising campaigns should therefore provide more information about the strategies and success probabilities of conservation measures. Similarly, people should be made aware of the importance and necessity of their financial support to implement effective measures. Since efficacy was identified in the present study as a significant factor influencing WTD, but not actual donations, future studies should test the extent to which efficacy may indirectly influence donations *via* the WTD.

A significant challenge for actual donation behavior seems to be in overcoming anticipated response barriers. Consistent with previous studies (Hermann and Menzel, 2013; Mankad et al., 2013; Bockarjova and Steg, 2014; Rainear and Christensen, 2017), we also found a negative influence of response barriers on the actual donations to insect conservation. Research indicates that response barriers have the largest negative effect on cost-intensive behaviors (Zhao et al., 2016). Accordingly, financial expenditures, appear to represent a high barrier to action. Many people may lack the confidence that their donated money will be invested in appropriate conservation measures. Therefore, fundraising campaigns should be transparent regarding the distribution of funds. Future studies should examine such inhibiting factors more closely to implement targeted strategies for overcoming response barriers in fundraising campaigns.

#### Knowledge

As predicted, we did not find evidence of knowledge as a predictor of WTD or actual donations toward insects in Germany. Similar to the present result, Onel and Mukherjee (2016) did not find any influence of factual knowledge about the environment on the WTD for different environmental conservation measures. Nevertheless, several studies have shown that knowledge can have a positive influence on attitudes and therefore on behavioral intentions (Schahn and Holzer, 1990; Kaiser and Fuhrer, 2003; Cornelisse and Sagasta, 2018). For example, Monge-Nájera (2017) found that a short natural history lecture on worms improved the attitudes toward these usually rather negatively perceived invertebrates. The present study indicates a small but significant positive correlation between knowledge and attitudes. Therefore, future studies should more closely examine the interaction effects of factual knowledge and attitudes on WTD and actual donations to insect conservation. Overall, it is crucial to acknowledge the knowledge that people in Germany appear to have about insects is rather low.

Furthermore, the inability of knowledge to explain the variance in WTD or actual donations could be because only factual knowledge about insects was tested. According to Frick et al. (2004), learning about what can be done about a problem (action knowledge) and knowledge about how these actions affect the environment (effectiveness knowledge) are essential factors influencing environmentally friendly behavior. Moreover, Cornelisse and Sagasta (2018) found that specific knowledge about the essential role of arthropods in ecosystems resulted in a higher conservation intention toward them. In particular, explicit messages about the useful functions of insects for human well-being, such as cleaning a local water source or pollinating a favorite fruit crop, could be effective (Schultz, 2011). Accordingly, the convergence of different types of knowledge about insects could influence the WTD and actual donations to insect conservation more positively. Overall, the results of this study imply that the communication of knowledge about insects in an educational context should go beyond pure factual knowledge.

#### Attitudes

We hypothesized that attitudes toward insects would influence WTD and actual donations to insect conservation in Germany. They were identified as the strongest predictor of WTD but did not impact actual donations. Numerous studies also found a significant relationship between positive attitudes and the WTD for biodiversity and species conservation (Kotchen and Reiling, 2000; Aldrich et al., 2006; Martín-López et al., 2007; Spash et al., 2009; Wilson and Bruskotter, 2009; Choi and Fielding, 2013; Zander et al., 2014). While in our study attitudes toward insects were rather positive compared to the midpoint of the scale, many authors point out that humans tend to have rather negative attitudes toward insects compared to other animal species, as they are often perceived as unaesthetic, disgusting, or dangerous (Kellert, 1993; Lorenz et al., 2014; Samways, 2018; Fukano and Soga, 2021; Prokop et al., 2021). Accordingly, Martín-López et al. (2007) detected a lower WTD for invertebrates than for mammals and birds. These findings suggest that measures to promote positive attitudes toward insects, seem to be of great importance for the WTD toward insect conservation.

In this context, future fundraising campaigns could highlight charismatic insect species, such as butterflies or bees, as flagship species (Oberhauser and Guiney, 2009). Alternatively, appealing characteristics of lesser-known species could be emphasized by, for example, using microphotography to highlight the unique colors and structures of insects (Stokes, 2006). Educational measures also play an essential role (Shipley and Bixler, 2017; Simaika and Samways, 2018), Monge-Nájera (2017) reported that a short lecture on the natural history of worms could cause a significant improvement of attitudes toward these usually negatively perceived animals. Additional studies showed that positive attitudes can be fostered by interactions between students and teachers (Wagler and Wagler, 2011), childhood experiences (Shipley and Bixler, 2019), or educational measures about the ecosystem services provided by insects (Stokes, 2006; Samways, 2018; Segerer and Rosenkranz, 2018; BMU, 2019; Cardoso et al., 2020). Increasing positive attitudes toward insects *via* these measures could consequently have a positive effect on the WTD to insect conservation.

The lack of a relationship between attitudes toward insects and actual donation behavior in this study may be present because only general attitudes toward insects were collected. According to Ajzen (2005) and Hini et al. (1995), attitudes related to the behavior to be executed are a particularly appropriate predictor for corresponding actions. Therefore, future studies should collect the specific attitudes of participants to the donation of money for insect conservation in Germany. Another useful approach could investigate the attitudes toward specific insect species which are visually presented to participants. For example, donation behavior toward attractive vs. unattractive and threatened vs. not threatened insects could be tested in an experimental set up using a 2 × 2 factorial design.

In the present study, we found that participants had rather positive attitudes towards insects (*M* = 3.46 on a five-point Likert scale). However, previous research suggests that insects are often evaluated negatively (e.g., Fukano and Soga, 2021; Prokop et al., 2021). The present findings could be explained by the fact that there are relatively few dangerous insects in Germany, as a result people may be less afraid of them. Furthermore, the sample may have been affected by self-selection. Due to the sampling method, participants may have already had a heightened interest in insects or nature. Our results on attitudes and knowledge also correspond with Prokop et al. (2009), who found that in countries where people perceive not much danger from insects, knowledge, and attitudes do not correlate strongly.

Finally, it should be noted that global attitudes toward insects are tending to develop positively due to people’s growing awareness of their ecological dependence on these animals (Samways, 2018). The rather positive attitudes toward insects of our participants may therefore also reflect a general change in attitudes toward insects in Germany. As such, this trend should be further investigated in future studies.

### Limitations of This Study

Regarding the representativeness of the study, the sociodemographic variables deviate slightly from the distribution of the population in Germany. Due to the requirements for participation in a panel study, no subjects under the age of 18 could be included, so the present study results only reflect the adult population. In addition, the participants could decide independently whether they wanted to participate in the survey. Due to this self-selection (Baur and Blasius, 2014), it is possible that primarily people with a generally higher interest in insects participated in the study. Moreover, it must be regarded critically that the WTD was assessed using only one item. If possible, future studies should assess behavioral intentions using multiple items.

## Conclusion

This study aimed to investigate the PMT constructs, knowledge, attitudes, and sociodemographic variables as possible factors influencing the WTD and actual donations toward insect conservation in Germany. Our results indicate that attitudes, severity, efficacy, educational level, and income have a positive influence on the WTD, while the actual donations could be predicted by gender, age, severity, and response barriers. In addition, a significantly positive influence of WTD on the actual donation behavior was found, but the WTD explained only a small part of the total variance. Overall, the PMT constructs were able to explain the largest part of the total explained variance in WTD and actual donations toward the conservation of insects in Germany.

Some implications for future awareness and fundraising campaigns can be derived from these results. In particular, people should be made more aware of the extent to which insects are endangered and the resulting consequences for humans, thus emphasizing their responsibility for nature and biodiversity. An effective strategy would be to convey that valuing and conserving insects is essential (Samways et al., 2020). At the same time, it is necessary to promote people’s self-efficacy and response efficacy to conserve insects. Therefore, future fundraising campaigns should educate people about the strategies and success probabilities of conservation measures. Moreover, the importance and necessity of their financial support needs to be emphasized. Especially measures promoting positive attitudes toward insects seem to be suitable to promote people’s WTD to the conservation of insects.

Furthermore, the results of this study have some implications for future research. Given the strong influence of positive attitudes toward insects on the WTD, future studies should examine more closely the extent to which different educational and awareness-raising measures can promote the formation of positive attitudes. Since the PMT constructs contributed significantly to the explanation of the WTD to insect conservation, future experimental studies could investigate more closely how the threat and coping assessment in the context of insect conservation could be positively influenced, for example, by giving specific information about the extent of insect mortality. Since some of the investigated variables were significant predictors of WTD but not of actual donations, mediator, or moderator effects between PMT constructs, knowledge, and attitudes should be analyzed more closely in the future. Furthermore, it is essential to investigate the relationship between intentions and behavior specifically and to identify inhibiting and promoting factors to derive context-specific implications for promoting real donations for insect conservation. This way the gap between conservation intentions and actual behavior could be better understood.

## Data Availability Statement

The raw data supporting the conclusions of this article will be made available by the authors, without undue reservation.

## Ethics Statement

Ethical review and approval was not required for the study on human participants in accordance with the local legislation and institutional requirements. The participants provided their written informed consent to participate in this study.

## Author Contributions

LD wrote the original draft of the manuscript. LD, MB, and AE performed the analyses, visualized the results, and revised the manuscript. JD and FF conceptualized the research and collected the data. FF supervised the project and revised the manuscript. All authors contributed to the article and approved the submitted version.

## Conflict of Interest

The authors declare that the research was conducted in the absence of any commercial or financial relationships that could be construed as a potential conflict of interest.

## Publisher’s Note

All claims expressed in this article are solely those of the authors and do not necessarily represent those of their affiliated organizations, or those of the publisher, the editors and the reviewers. Any product that may be evaluated in this article, or claim that may be made by its manufacturer, is not guaranteed or endorsed by the publisher.
